# Challenges in Diagnosing Hepatic Sarcoidosis: A Case Report

**DOI:** 10.7759/cureus.74267

**Published:** 2024-11-22

**Authors:** Adil Zegmout, Ayman El Farouki, Aniss Rafik, Amine Elwardi, Mohamed Essarghini

**Affiliations:** 1 Faculty of Medicine and Pharmacy, Hassan II University, Casablanca, MAR; 2 Pulmonology Department, Hassan II Military Hospital, Laayoune, MAR; 3 Radiology Department, Hassan II Military Hospital, Laayoune, MAR; 4 Pulmonology Department, Mohamed V Military Hospital, Rabat, MAR; 5 Pathology Department, Annasr Pathology Center, Laayoune, MAR; 6 Visceral Surgery Department, Hassan II Military Hospital, Laayoune, MAR

**Keywords:** diagnostic challenges, differential diagnosis, granulomatous disease, hepatic sarcoidosis, multidisciplinary approach

## Abstract

Hepatic sarcoidosis is rare, and its similarity to liver metastases complicates the diagnosis. This mimicry requires a thorough diagnostic investigations to exclude neoplasia and other granulomatous diseases, particularly tuberculosis. A 36-year-old male presented with a two-month history of right hypochondrial tenderness, anorexia, asthenia, and weight loss. Clinical examination showed hepatomegaly and splenomegaly. A CT scan revealed non-compressive mediastinal lymphadenopathy and nodular hepatosplenomegaly. Liver function tests indicated cholestasis. The differential diagnosis included lymphoma, metastases, tuberculosis, and sarcoidosis. Biological assessments were normal except for elevated serum angiotensin-converting enzyme (120 U/L) and hypoalbuminemia with polyclonal hypergammaglobulinemia. A CT-guided liver biopsy showed non-caseating granulomas. Tuberculosis tests, including acid-fast bacilli, GeneXpert (Cephei Corp., Sunnyvale, CA) cultures, and Quantiferon (QIAGEN, Venlo, Netherlands) were negative. Multidisciplinary discussion supported sarcoidosis based on clinical, biological, radiological, and histological findings. Hepatic sarcoidosis can mimic conditions like tuberculosis and metastases. Accurate diagnosis is crucial for appropriate management, highlighting the need for awareness of atypical presentations.

## Introduction

Sarcoidosis is a multisystem granulomatous disease of unknown aetiology, characterized histologically by the presence of non-caseating epithelioid cell granulomas [1]. Sarcoidosis mainly affects the lungs and intrathoracic lymph nodes, but can also affect many other organs, including the skin, eyes, heart, spleen, bone marrow and liver. Hepatic sarcoidosis, a relatively rare manifestation, affects around 11% of patients with sarcoidosis [2]. This rarity requires increased clinical vigilance and consideration of liver involvement in the differential diagnosis of sarcoidosis.

The diagnosis of hepatic sarcoidosis is particularly difficult due to its clinical and radiological similarities with liver metastases, which raises a problem of differential diagnosis [3]. Also, imaging techniques can reveal multiple hepatic nodules that mimic secondary metastatic localisations [4]. This similarity requires thorough diagnostic investigations to exclude neoplasia and other granulomatous diseases, particularly tuberculosis.

Misdiagnosis can have serious consequences. Patients will be exposed to unnecessary invasive procedures, surgery interventions and systemic chemotherapy, which may result in adverse effects. However, rapid and accurate diagnosis is imperative to guide appropriate therapeutic strategies, avoid unnecessary treatment and improve patient prognosis.

In this report, we highlight the clinical and radiological aspects of a case of hepatic sarcoidosis that presented as multiple disseminated hepatic nodules, initially suspected as metastatic disease on computed tomography imaging, and subsequently confirmed as sarcoidosis via histopathological analysis. This case also underscores the difficulty of differential diagnosis with tuberculosis, which is common in our country.

## Case presentation

A 36-year-old patient presented with a two-month history of right hypochondrial tenderness, anorexia, asthenia, and weight loss. The patient did not have a significant past medical history nor a history of smoking, alcoholism, or known neoplasia. He had never been treated for tuberculosis and has no recent exposure to tuberculosis. On clinical examination, the patient was conscious, and respiratory and haemodynamic parameters were stable. There was no pallor or jaundice. The abdominal examination revealed a hepatomegaly and splenomegaly. There was no palpable peripheral lymphadenopathy and the ophthalmological examination was normal with no evidence of uveitis.

**Figure 1:**
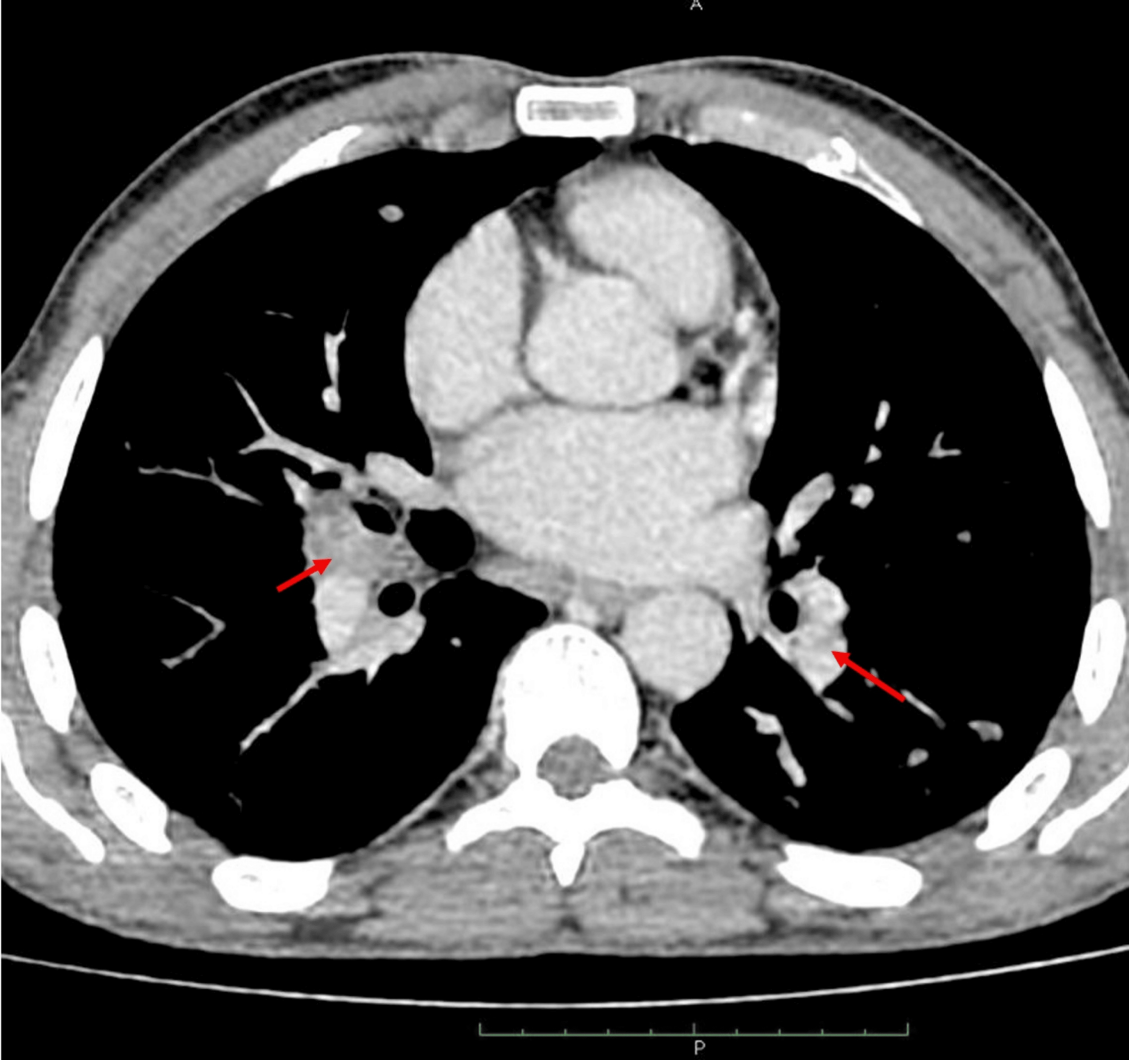
Thoracic CT scan showed multiple bilateral, non-compressive and non-necrotic mediastinal lymphadenopathy (red arrows).

A thoracic CT scan showed multiple bilateral, non-compressive and non-necrotic mediastinal lymphadenopathy (Figure 1), including stations 3A, 2R, 4R, 4L, 6, and 10R, with the largest in station 4R (12 mm) and 10R (18 mm).

**Figure 2:**
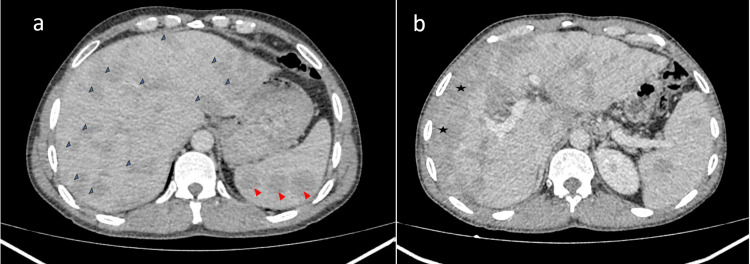
(a) Abdominal CT scan shows an enlarged liver with multiple hypodense nodules (the largest are marked by blue arrows). The spleen contains multiple hypodense nodules (red arrows). (b) Some liver nodules are confluent, forming irregular, ill-defined hypodense areas (stars).

Nodular hepatosplenomegaly was present in the abdominal CT scan. The liver was enlarged and contained multiple hypodense nodules, the largest measuring 55 x 26 mm in segment III (Figure 2a). Some nodules were confluent forming irregular, ill-defined and hypodense areas, with capsular retraction on peripheral lesions (Figure 2b). The spleen measured 16 cm and contained multiple hypodense nodules, the largest measuring 35 x 32 mm (Figure 2a).

The differential diagnosis considered included malignant haematological diseases such as lymphoma, hepatic and splenic metastases from solid tumours, tuberculosis, or sarcoidosis.

Liver function tests indicated cholestasis without jaundice: ASAT at 39 U/l, ALAT at 45 U/l, GGT at 240 U/l, alkaline phosphatase (ALP) at 451 U/l, bilirubin at 8.4 mg/l, and prothrombin at 81%. The biological assessment was normal, including the hemogram and the levels of tumour markers (CA19-9, CEA, NSE, AFP, PSA, and thyroglobulin). Calcium and phosphate levels, as well as urinary calcium, were normal. However, the serum angiotensin-converting enzyme was elevated at 120 U/L. Electrophoresis indicated hypoalbuminemia with polyclonal hypergammaglobulinemia and decreased beta-1 globulins. The 24-hour proteinuria level was normal (<0.07 g/L). An accessory salivary gland biopsy showed minor chronic sialadenitis (Schisholm and Masson grade I) without granulomas. Flexible bronchoscopy revealed no visible abnormalities. Multiple endobronchial biopsies were taken at different bronchial levels, showing chronic nonspecific inflammation without granulomas. The bronchoalveolar lavage analysis revealed a normal cell count. Pulmonary function tests were normal, with a vital capacity of 98% and a total lung capacity of 101%.

**Figure 3:**
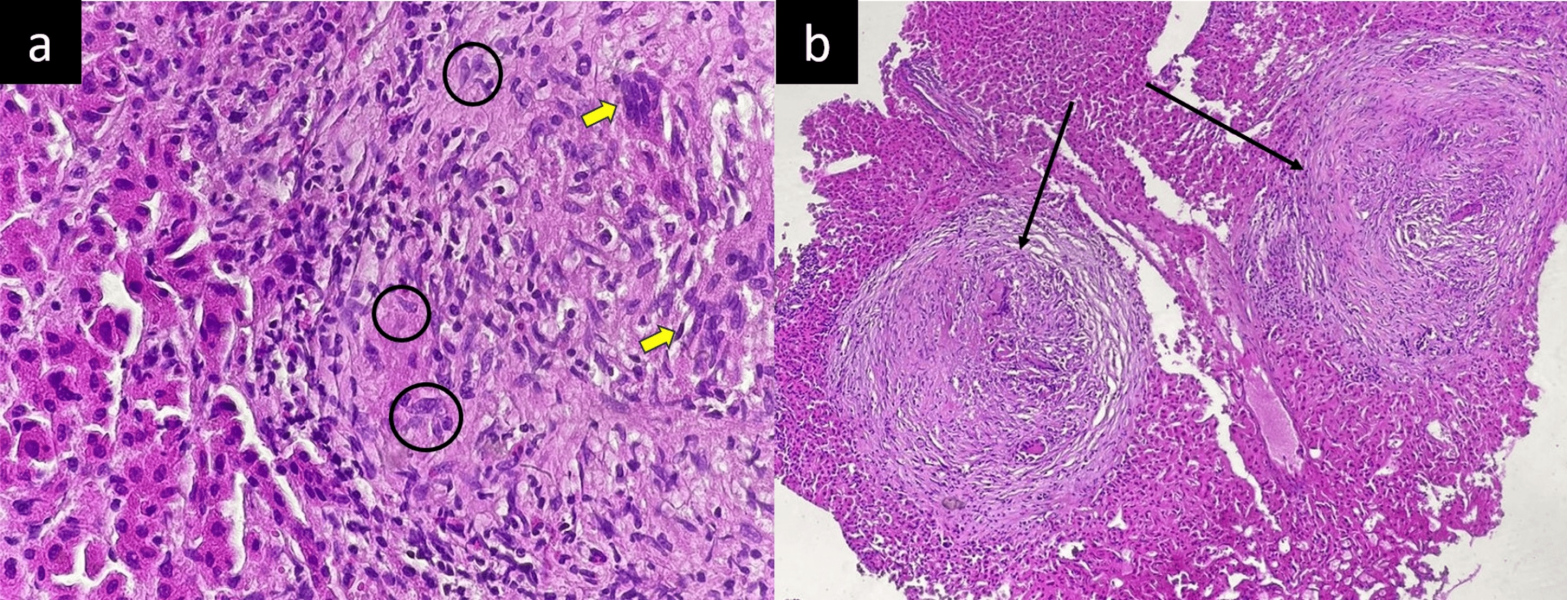
(a) Histopathological findings of a liver biopsy (high magnification): granuloma composed of multinucleated giant cells (yellow arrows) and epithelioid cells (circles) without necrosis. (b) Histopathological findings of a liver biopsy (low magnification): hepatic parenchyma with caseating granulomas (black arrows) morphologically compatible with a sarcoid-like reaction.

Histological examination of a CT-guided liver biopsy revealed epithelioid cell granulomas with multinucleated giant cells (Figure 3a), consistent with non-caseating granulomas (Figure 3b). Given this granulomatosis and to exclude tuberculosis, additional tests were performed. Acid-fast bacilli were not detected in the expectorated samples. Moreover, GeneXpert results (Cephei Corp., Sunnyvale, CA) from both bronchoalveolar lavage and hepatic biopsy did not reveal the presence of *Mycobacterium tuberculosis*. The cultures from various samples remained negative after two months of incubation in a specific medium. Additionally, the interferon-gamma release assay (Quantiferon, QIAGEN, Venlo, Netherlands) was negative.

After a multidisciplinary discussion, the diagnosis of sarcoidosis appears most probable based on clinical, biological, radiological, and histological arguments in a patient without any significant medical history or risk factors.

**Figure 4:**
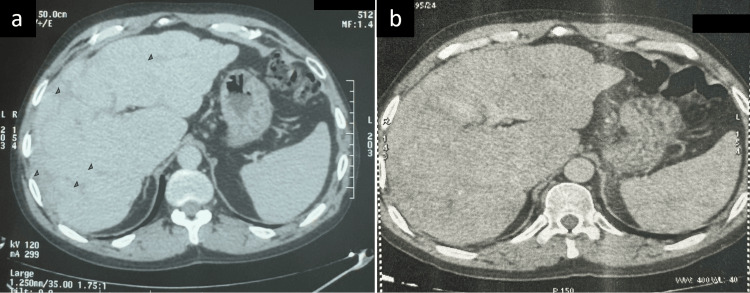
(a) Control abdominal CT scan at three months shows a significant reduction in hepatic nodules (arrows) and the disappearance of splenic nodules. (b) Control abdominal CT scan at 12 months shows regression of hepatic and splenic lesions.

Treatment was started with corticosteroids at an initial dose of 0.5 mg/kg/day (40 mg/day). After three months of treatment, clinical symptoms improved significantly, liver function tests and angiotensin-converting enzyme levels normalized, and a control CT scan showed a significant reduction in hepatic nodules, and disappearance of splenic nodules (Figure 4a). Given this favourable response, corticosteroids were gradually tapered to a maintenance dose of 7.5 mg/day. An abdominal CT scan performed at 12 months of treatment showed regression of hepatic and splenic lesions (Figure 4b). The duration of treatment was 18 months. Follow-up included imaging studies, pulmonary function tests, monitoring of liver function tests and angiotensin-converting enzyme levels, all of which remained normal throughout the treatment period. The favourable response to corticosteroid therapy supports the diagnosis of sarcoidosis in this patient.

## Discussion

The differential diagnosis of hepatic nodules is complex, including metastatic cancer and benign conditions such as sarcoidosis or tuberculosis [3]. Patients with hepatic sarcoidosis may exhibit various nonspecific symptoms, including abdominal pain and weight loss, or may be asymptomatic, with incidental findings on imaging performed for other reasons [5].

Imaging studies such as positron emission tomography (PET), magnetic resonance imaging (MRI) and computed tomography (CT) are important in the initial assessment but often cannot definitively distinguish hepatic sarcoidosis from metastatic lesions due to their similar radiologic appearance and disseminated nature of the lesions [6], as highlighted our cases. The way that nodules enhance on imaging after contrast administration can be similar in both sarcoidosis and metastases, particularly if the nodules are hypoenhancing [4]. The typical imaging findings of hepatic sarcoidosis can vary considerably. Common radiological characteristics include homogeneous hepatomegaly or multiple nodules scattered throughout the parenchyma [7]. These nodules are usually well-defined and can vary in size, potentially mimicking liver metastases [4]. Despite these similarities, certain imaging features might suggest sarcoidosis rather than metastasis, such as the absence of mass effect on adjacent structures, the presence of adenopathy (particularly hilar) and the presence of splenic nodules [7]. Biopsy remains the cornerstone of diagnosis when imaging is inconclusive [1], as evidenced by our patient, whose lesion biopsy revealed non-caseating granulomas indicative of sarcoidosis.

The diagnosis of hepatic sarcoidosis can be particularly complex; not only because it must be differentiated from hepatic metastases, as previously discussed, but also from tuberculosis. Both hepatic sarcoidosis and hepatic tuberculosis can present with similar symptoms and liver function test abnormalities, and both conditions can lead to the formation of granulomas in the liver [1]. Differentiating between the two diseases is further complicated in regions with a high prevalence of tuberculosis like Morocco. In 2022, 30,503 cases of tuberculosis in all forms were reported, which corresponds to an incidence of 82 per 100,000 inhabitants [8]. A thorough clinical history and a review of any known exposure to tuberculosis are important. Testing for tuberculosis, such as a tuberculin skin test or interferon-gamma release assays, and assessment for evidence of caseation necrosis in biopsy specimens, which is characteristic of tuberculosis but not of sarcoidosis, may also help in the differentiation [9]. The culture of biopsy fragments on a specific media is necessary for the identification of *Mycobacterium tuberculosis*. The GeneXpert test on biopsy fragments can also help in the diagnosis of hepatic tuberculosis, with the advantage of being rapid and specific [10].

Ultimately, the diagnosis of hepatic sarcoidosis is often one of exclusion after tuberculosis, metastases, and other causes of granulomatous liver disease have been carefully considered and ruled out. The diagnostic approach often involves a combination of clinical assessment, imaging studies, laboratory tests, histological examination and response to therapy [5]. Despite these methods, the definitive diagnosis of hepatic sarcoidosis can remain elusive without a detailed and thorough investigative process. This can sometimes lead to invasive procedures such as liver biopsy or even surgical resection for definitive diagnosis [3]. As was the case with our patient, CT-guided liver biopsies were necessary due to the radio-clinical presentation.

Treatment of hepatic sarcoidosis usually depends on the severity of the symptoms and the extent of liver involvement [2]. Many patients with hepatic sarcoidosis may be asymptomatic and not require treatment. However, if the liver function is significantly affected or symptoms are present, treatment options may include corticosteroids as a first-line therapy. In cases where corticosteroids are ineffective or intolerable, alternatives include immunosuppressive agents, and TNF-alpha inhibitors may be used [5].

## Conclusions

Hepatic sarcoidosis can manifest in various patterns. Diagnosis can be challenging and may mimic other conditions, including tuberculosis and hepatic metastases. This case highlights the importance of a comprehensive diagnostic approach that includes clinical assessment, imaging studies, laboratory tests and histological examination. By considering all available information and ruling out other potential causes, a multidisciplinary discussion can lead to an appropriate treatment plan for patients with hepatic sarcoidosis. A liver biopsy should be performed promptly and without hesitation if a definitive diagnosis is difficult to establish. Increased awareness and understanding of the atypical presentations of hepatic sarcoidosis are crucial for accurate diagnosis and appropriate management.
